# INSIGHTS GAINED FROM A VIRTUAL INTERGENERATIONAL YOGA INTERVENTION DURING COVID-19

**DOI:** 10.1093/geroni/igae098.4225

**Published:** 2024-12-31

**Authors:** Nancy Martens, Debra Schutte

**Affiliations:** University of Michigan, Flint, Michigan, United States; Wayne State University, Detroit, Michigan, United States

## Abstract

Studies focused on social isolation, intergenerational, and virtual interventions revealed lack of rigor (Cohen-Mansfield et al., 2018, Conn & Grove, 2019; Chipps et al., 2017). The Shine Through2 pilot study aimed to determine feasibility and acceptability of an intergenerational yoga intervention delivered virtually, twice weekly, for six weeks. The Shine Through2 project was tailored for populations who may be experiencing social isolation. The project was developed as a novel community and university collaboration pilot and is a starting point for establishing partnerships aimed to improve older adult and college student health, and subsequently community health. The project included 23 older adults and 12 college students. The data suggest older adults and college students found this virtually - delivered socialization and yoga intervention feasible and acceptable. Insights gained from the project include: 1) Pre-screening for social isolation and depression are impactful assessment modalities, 2) Utilizing a Senior Certified Yoga Instructor added (necessary) expense and scalability challenges, 3) Sampling bias can be overcome with intentional recruitment strategies 4) Specialized yoga programming may be cost-prohibitive, 5) Intergenerational component challenging to deliver virtually, 6) Study design did not allow direct comparison of efficacy between older adults and college students, and 7) Provision of community yoga resources was limited by COVID-19. Based on the results explored in ShineThrough2, we conclude that socialization and yoga interventions can be used to improve health outcomes including measures of social isolation, depressive symptoms and meaning in life.

